# Control of a Robot Arm Using Decoded Joint Angles from Electrocorticograms in Primate

**DOI:** 10.1155/2018/2580165

**Published:** 2018-10-18

**Authors:** Duk Shin, Hiroyuki Kambara, Natsue Yoshimura, Yasuharu Koike

**Affiliations:** ^1^Tokyo Polytechnic University, Tokyo, Japan; ^2^Tokyo Institute of Technology, Tokyo, Japan

## Abstract

Electrocorticogram (ECoG) is a well-known recording method for the less invasive brain machine interface (BMI). Our previous studies have succeeded in predicting muscle activities and arm trajectories from ECoG signals. Despite such successful studies, there still remain solving works for the purpose of realizing an ECoG-based prosthesis. We suggest a neuromuscular interface to control robot using decoded muscle activities and joint angles. We used sparse linear regression to find the best fit between band-passed ECoGs and electromyograms (EMG) or joint angles. The best coefficient of determination for 100 s continuous prediction was 0.6333 ± 0.0033 (muscle activations) and 0.6359 ± 0.0929 (joint angles), respectively. We also controlled a 4 degree of freedom (DOF) robot arm using only decoded 4 DOF angles from the ECoGs in this study. Consequently, this study shows the possibility of contributing to future advancements in neuroprosthesis and neurorehabilitation technology.

## 1. Introduction

Brain machine interface (BMI) is a communication tool for quadriplegia between the brain and external devices such as a robot [1]. Since electroencephalography (EEG) has high temporal resolution, prominent EEG-based studies have been used in various paradigms, such as a computer cursor control [2], direction intention of hand movements [3, 4], a P 300 speller device [5], and neurofeedback systems for rehabilitation [6–9]. These noninvasive methods are useful for classification of movement direction or intention. The prediction of time-varying trajectories, however, is very difficult because of insufficient spatial resolution and low signal-to-noise ratio.

After the first electrocorticogram- (ECoG-) based BMI succeeded in one-dimensional cursor control in humans [10], it has come into the spotlight as an alternative recording approach for less invasive BMIs because ECoG signals could offer higher spatial resolutions than the classical scalp EEG signals. ECoG signals have also shown direct potential as a stable in long-term recording method [1, 11–14]. Many studies using ECoG have already succeeded in the classification of movement direction [15, 16], grasp type [17], and the prediction of hand trajectory [11–13, 18]. Our previous studies based on the brain rhythmic bands with SLiR algorithm also have been reported to predict muscle activities and arm trajectories from ECoG signals [13, 14, 19–22].

Despite these successes, however, there still remain works for the realization of ECoG-based BMIs. The brain and CNS system allows skillful manipulation of the body to interact with the external environment. This sophisticated and flexible operation involves intrinsic (kinetic) information such as force and stiffness, and as well as extrinsic (kinematic) information such as desired angle or velocity. The neuromuscular system naturally modulates mechanical stiffness and viscosity in order to obtain proper interaction force to the environments. Since stiffness, viscosity, and force change with our muscle activation in order to interact with environments, decoding muscle activities, as well as joint angles, are important components for realizing neuroprosthesis capable of controlling interaction force or stiffness. Although many studies have achieved to decode arm trajectory, a few studies have demonstrated that muscle activity could be decoded from spike signals [23–25], EEG [26, 27], and ECoG [19, 22].

**Figure 1:**
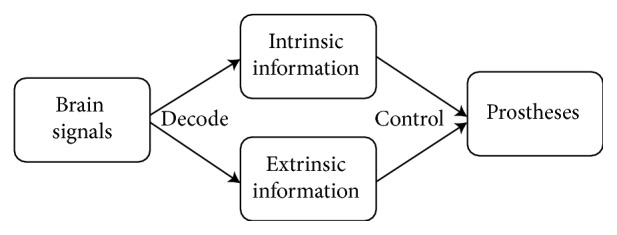
Schematic outline of a proposed neuroprosthesis.

The purpose of this study is to propose a basis concept for a neuromuscular BMI system. The schematic outline of this concept is shown in Figure 1. A well-trained Japanese monkey performed a series of reaching, grasping, pulling, and releasing movements. We simultaneously recorded 16 ECoG signals of the primary motor cortex (Ml) and 12 electromyography (EMG) signals in the right arm. We also measured and calculated joints angle using a 3D marker tracking system. We decoded three joint angles for the shoulder, elbow joint angle, and muscle activities from ECoG signals using sparse linear regression (SPR). Our results indicate that ECoG signals from primary motor cortex involves both intrinsic (kinetic) and extrinsic (kinematic) information. We could also predict multiple muscle activities (intrinsic) and joint angles (extrinsic) from ECoG signals simultaneously. In addition, we controlled a 4 degree of freedom (DOF) robot arm using decoded joint angles and muscle activation in offline simulation.

## 2. Material and Methods

### 2.1. Behavioral Task

All experimental procedures were performed in accordance with the Guidelines for Proper Conduct of Animal Experiments of the Science Council of Japan and approved by the Committee for Animal Experiment at the National Institutes of Natural Sciences (Approval No. 11A157). The animal welfare and steps taken to ameliorate suffering were in accordance with the recommendations of the Weatherall report, “The use of nonhuman primates in research.”

**Figure 2:**
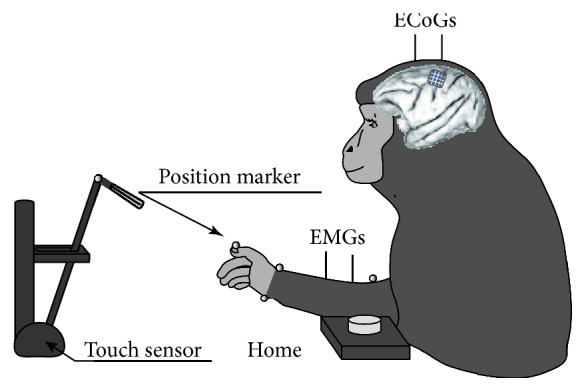
Behavioral task. Monkeys performed sequential right arm and hand movements, in a 3D workspace. During the task, ECoG, EMG signals, and marker positions were recorded simultaneously.

We note that we reused our database reported in our previous work [19] for verifying the possibility of realizing neuromuscular BMI device. We explain briefly experimental setup in this paper as follows. A Japanese macaque (female, at 4.7 kg) was trained to perform reaching and grasping tasks with the right hand as shown in Figure 2. First, the monkey placed her hand on a home button located in front of the chair. Second, the monkey tried to reach for the knob after a “go” cue was given in a beep sound. Third, the monkey then had to pull the knob and release. When the monkey successfully pushed the home button and pulled the knob to the required displacement (6 cm), it received a juice reward. We extracted continuous data (total length; 500 s) from our database to control the robot arm. These data involved a total number of 248 trials. Each trial duration averages and standard deviations (STD) were 1.16 ± 0.29s.

### 2.2. Data Recording

**Figure 3:**
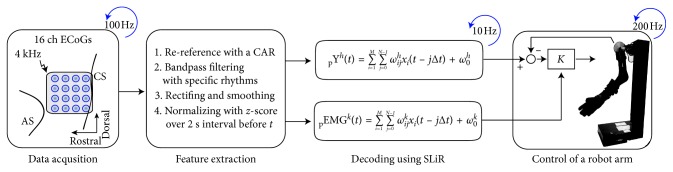
Algorithm flowchart of control of a robot arm using decoded joint angles and muscle activation from ECoG signals.

A platinum ECoG array (Unique Medical Corporation, Tokyo, Japan), which had 16 (4 × 4 grid) channel electrodes was implanted over the left primary motor cortex (M1) as shown in Figures 2 and 3.

EMG signals of the right forelimb muscles were recorded from chronically implanted pairs of multistranded stainless steel wires (Cooner Wire, Chatsworth, CA, USA). They were subcutaneously tunneled to the following target muscles: adductor pollicis (AP), abductor pollicis longus (APL), flexor digitorum profundus (FDP), and extensor digitorum communis (EDC) for hand muscles; flexor carpi ulnaris (FCU), and extensor carpi radialis (ECR) for wrist muscles; brachioradialis (BRA) and triceps lateral head (TRA) for elbow muscles; biceps long head (BIL) and triceps long head (TRO) for shoulder-elbow double joint muscles; and pectoralis major clavicular head (PECM) and deltoid clavicular part (DELP) for shoulder muscles. ECoG and EMG signals were sampled at 4 kHz.

The 3-D positions were recorded using the optical motion capture system (Eagle digital system; Motion Analysis Corporation, Santa Rosa, CA). The system used twelve infrared cameras operating at 200 frames/s to track the positions of multiple reflective markers (4 mm-diameter spheroids). A total of fourteen markers were attached to the right arm of the monkey from the shoulder to the fingers. The motion data were down-sampled to 100 samples per second.

### 2.3. Preprocessing of ECoG and EMG Data

ECoG signals were preprocessed with our previously proposed method [19]. First, the signal data were re-referenced with a common average reference. Second, each ECoG signal was divided into nine frequency bands (*δ*: 1.5–4 Hz; *θ*: 4–8 Hz; *α*: 8–14 Hz; *β*1: 14–20 Hz; *β*2: 20–30 Hz; *γ*1: 30–50 Hz; *γ*2: 50–90 Hz; *γ*3: 90–120 Hz; and *γ*4: 120–150 Hz) using fourth-order bandpass Butterworth filters. Second, the bandpass filters split 16-channel ECoG signals into nine band-passed signals to produce *M* channels of bandpass filtered signals ${\hat{x}}_{i}\left(t\right)$. Third, these band-passed signals were digitally rectified and smoothed with a Gaussian filter (width: 0.1 s, *σ*: 0.04 s). Fourth, the obtained signals at time *t* were normalized to the standard *z*-score as follows:

(1) $$\begin{array}{c} x_{i}\left(t\right)=\frac{{\hat{x}}_{i}\left(t\right)-{\hat{\mu }}_{i}}{{\hat{\sigma }}_{i}},\;\left(i=1,2,3,\ldots ,M\right), \end{array}$$

where ${\hat{\mu }}_{i}$ and ${\hat{\sigma }}_{i}$ are the mean and the standard deviation of ${\hat{x}}_{i}$, over a 2 s interval before the time *t*. Finally, the signals were down-sampled to 100 Hz to match the motion data.

EMG signals were rectified and passed through a fourth-order lowpass filter with a cutoff frequency of 4 Hz and further down-sampled to 100 Hz, resulting in muscle activation [28, 29].

### 2.4. Prediction of Muscle Activation and Joint Angles from ECoG Signals

We used the Variational Bayesian Sparse Regression toolbox [30] to decode muscle activation and joint angles. The decoded muscle activation pEMG^*k*^(*t*) and joint angles pY^*h*^(*t*) at time *t*, are described as

(2) $$\begin{array}{c} {\text{pEMG}}^{k}\left(t\right)=\overset{M}{\underset{i=1}{\sum }}\overset{N-1}{\underset{j=0}{\sum }}\omega_{ij}^{k}x_{i}\left(t-j\Delta t\right)+\omega_{0}^{k},\\ {\text{pY}}^{h}\left(t\right)=\overset{M}{\underset{i=1}{\sum }}\overset{L-1}{\underset{j=0}{\sum }}\omega_{ij}^{h}x_{i}\left(t-j\Delta t\right)+\omega_{0}^{h}, \end{array}$$

where *ω*_*ij*_^*k*^ and *ω*_*ij*_^*h*^ are the weight coefficient of the *k*-th muscle and *h*-th joint angle for the *i*-th signal source at a delay time *j*Δ*t*, *ω*_0_^*k*^ and *ω*_0_^*h*^ are the bias terms, *x*_*i*_(*t*) is the *i*-th ECoG source at time *t*, and Δ*t* is a discrete-time step-size of 20 ms. The muscle activity at time *t* was predicted using 10 time points (*N* = 10) starting 200 ms before the target time *t*. The joint angles at time *t* were predicted using 25 time points (*L* = 25) starting 500 ms before the target time *t*.

### 2.5. Analysis

Accuracy of the predictions was evaluated using a 5-fold cross validation. The entire 500 s of experiment data were divided into two parts, 400 s of training data and 100 s of test data. We calculated the coefficients of determination (*R* ^2^) to evaluate the similarity between actual and predicted muscle activities. Accuracy was also evaluated using normalized root-mean-square error (*n*RMSE) between actual and predicted.

### 2.6. Robot Arm

We specially designed a life-sized robot arm for monkey to reproduce the movements of arm as shown in Figure 3. For the purposes of this study, the robot arm is modeled as a four DOF kinematic linkage, consisting of two links i.e., upper arm and forearm along with the hand and two joints i.e., shoulder joint and elbow joint, with a fixed wrist joint. The shoulder joint has 3 DOFs (S1: abduction/adduction; S2: flexion/extension; S3: rotation), and the elbow is simplified as a revolute joint with 1 DOF (E1: flexion/extension). The upper arm and forearm are driven by the brushless DC servo motors with 512 ppr encoder (Faulhaber Motors: 2342S024CR). The PID controller is implemented on the base, and basic trajectory tracking tasks were executed in joint space.

We also made an offline simulator. We made ECoG database at a 100 Hz sampling time for the simulator. The simulator reads 10 time points of ECoG data per cycle. We used first-in-first-out (FIFO) method with 2 s data length. The simulator calculates the desired joint angles and sends them to the control computer using TCP/IP protocol. Then, the control computer can calculate and update *θ*_ref_ in the PID controller on the robot arm with at a 200 Hz sampling rate (Figure 3):

(3) $$\begin{array}{c} \theta_{\text{ref}}=K\left(\theta_{\text{des}}-\theta_{\text{pre}}\right), \end{array}$$

where *θ*_des_ and *θ*_pre_ are the desired angle and the present angle and *θ*_ref_ is input value for robot arm. *K* gain should be controlled by the intrinsic information i.e., stiffness. We used an index of muscle cocontraction around the joint (IMCJ) instead of stiffness. Osu et al. reported the linear relationship between IMCJ and joint stiffness measured by the perturbation [29, 31]. We defined shoulder gain *K*_s_(*t*) and elbow gain *K*_e_(*t*) as follows:

(4) $$\begin{array}{c} K_{\text{s}}\left(t\right)=\underset{k}{\sum }\left|\beta_{\text{s}}\right|{\text{pEMG}}^{k}\left(t\right),\left(k=1,2,3,5\right),\\ K_{\text{e}}\left(t\right)=\underset{k}{\sum }\left|\beta_{\text{e}}\right|{\text{pEMG}}^{k}\left(t\right),\left(k=3,4,5,6\right), \end{array}$$

where *β*_s_ and *β*_e_ are constant gain and adjusted manually for stable movements.

## 3. Results

### 3.1. Prediction of Muscle Activation

**Figure 4:**
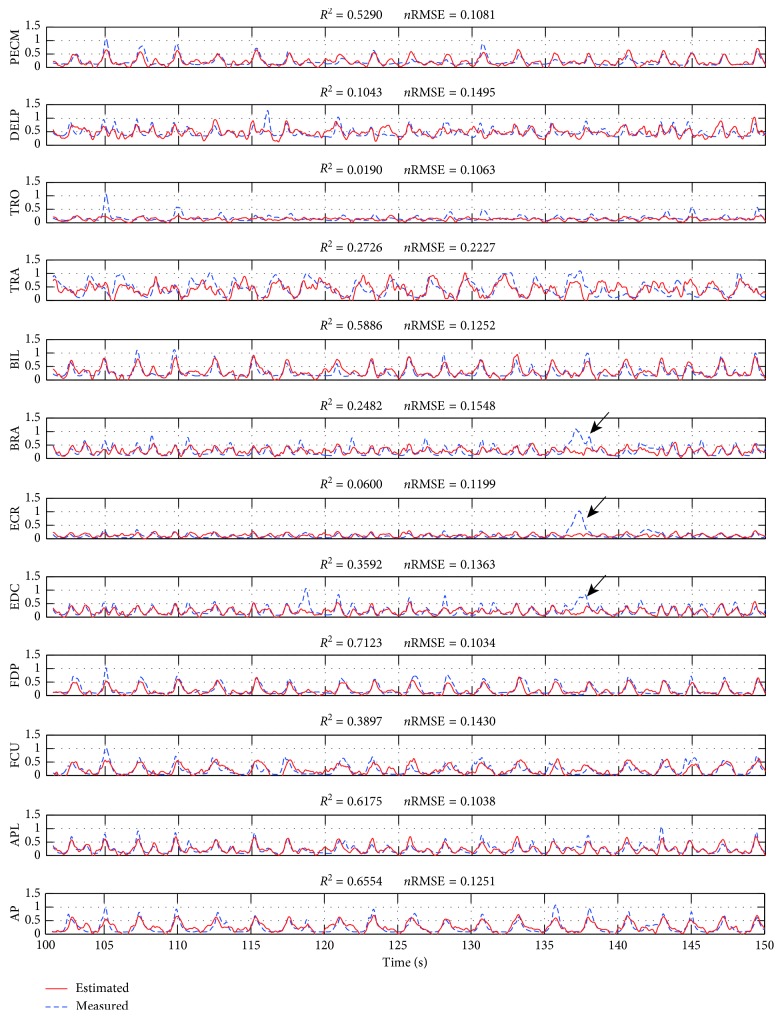
Example of typical muscle prediction. We show the result over 50 s of test data using 16 ECoG electrodes. *R* ^2^ values and *n*RMSE for the comparison between predicted (red solid) and observed (blue dotted) muscle activation are also shown.

We applied the decoding model to continuous test data. One typical example of continuous prediction is shown in Figure 4., where the prediction was stable even for repetitive trials over 100 s. We note that the results showed the similar pattern in the agonistic muscles and also even the antagonistic muscles such as TRO, ECR, and EDC. The arrows shown in Figure 4 indicated local difference between measured and estimated EMGs. These differences have appeared occasionally in this work. This measuring noise might be a result from the crosstalk among the EMG electrodes or the wobbling electrode during the monkey's motion. Although the results involved some failures, these results clearly show that the proposed method could realize neuromuscular BMI system in an online fashion. In the results of the 5-cross validation, means and standard deviation of *R* ^2^ and *n*RMSE for each muscle ranged from 0.0199 ± 0.0062 (ECR) to 0.6333 ± 0.0033 (FDP) and 0.1303 ± 0.0053 (APL) to 0.1825 ± 0.0098 (FCU) as shown in Table 1. The median and standard error of *R* ^2^ and *n*RMSE were 0.3765 ± 0.0164 and 0.1527 ± 0.0028, respectively. In the case of TRO, *R* ^2^ value was worse but *n*RMSE was good among muscle prediction. It might be the reason that the hardly used muscle such as TRO had small range of activation. The bold numbers in Table 1 show the best value among the test data.

### 3.2. Prediction of Joint Angle and Robot Control

**Figure 5:**
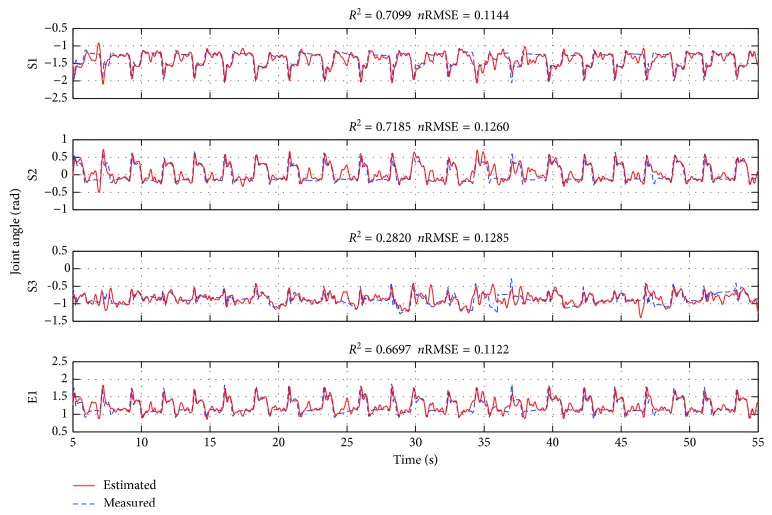
Example of typical joint angle prediction for test data using 16 ECoG electrodes. *R* ^2^ values and *n*RMSE for the comparison between predicted (red solid) and observed (blue dotted).

We also predicted joint angles from the ECoG signals with the SPR model. The desired angles calculated from offline simulation showed good correlation with the actual angles of movements as shown in Figure 5. In the results of the 5-cross validation, means and standard deviations of *R* ^2^ and *n*RMSE of angle prediction between the measured angles and the predicted ones were from −0.0333 ± 0.3547 (S3) to 0.6359 ± 0.0929 (S2) and from 0.1261 ± 0.0111 to 0.1596 ± 0.0286, respectively (Table 2). The median and standard error of *R* ^2^ and *n*RMSE were 0.6171 ± 0.1415 and 0.1373 ± 0.0083, respectively. The bold numbers in Table 2 also show the best value among the test data. These results suggest that the arm movement can be estimated from ECoG signals. The accuracy values of S1 (shoulder abduction/adduction), S2 (shoulder flexion/extension), and E1 (elbow flexion/extension) were higher than those of S3 (rotation). It could not affect the robot control because the range of shoulder rotation had small. (see also video S1 about the robot arm).

## 4. Discussion

Since ECoG signal is the origin of EEG signals, we tried to divide ECoG signals into specific brain rhythmic bands based on the traditional EEG studies. In addition, Chen et al. [13] also reported that the brain rhythmic based method produced the same or better results than the nonphysiological fractionized frequency method. The delta and gamma bands were superior to the other bands in this study. Our previous studies [15–17, 19, 20, 27] also reported that delta and gamma bands have plentiful information to estimate the trajectories and force. However, the weights of all frequency bands disappeared after applying the SPR algorithm. This phenomenon stood out in intrinsic (kinetic) prediction. This might indicate that all sensorimotor rhythms of ECoG are needed to predict EMG signals and angles.

Current rehabilitation robots can perform sophisticated operations including stiffness control [32, 33]. The human musculoskeletal system has stiffness and viscosity properties essential to interaction with our surroundings. The perturbation method has been used to measure stiffness of the human arm with a manipulator. Since the results are the averages of many experimental trials, stiffness could not be measured in time series. There are some trials that estimate stiffness using EMG signals because stiffness changes with muscle activation. Osu and Gomi tried to rebuild joint stiffness from EMG signals using conversion factors to match the EMG to the measured stiffness using PFM [34]. Osu et al. [31] consequently proposed IMCJ that was defined as the summation of absolute values of muscle quasitension. These earlier models used parameters or “gains” with no physiological basis, thus compromising constructional validity. Our previous studies suggested the myokinetic (Mykin) model which can estimate the angle, torque, and stiffness of joints from muscle activities [25, 29, 33]. In this study, we used IMCJ instead of Mykin model, because Mykin model should need the parameter calibration from the relationship between joint torque and muscle activation. Nonetheless, we could control the robot arm using both angles (extrinsic) and IMCJ (intrinsic). Therefore, decoding muscle activity and joint angles is an important component for realizing BMI systems capable of controlling interaction.

## 5. Conclusion

This study describes the prediction muscle activities and joint angles from ECoG signals. We displayed a novel attempt to control the 4 DOF robot arm using the decoded joint angles and muscle activation for neuromuscular BMI system. This approach offers important insight regarding the presence of ample information in ECoG signals to control a neuro-muscular prosthesis that behaves like a human arm. The results clearly demonstrated that muscle activities and joint angles could be predicted in time series from ECoG signals simultaneously, whereas previous ECoG-based studies have reported the classification of movement direction or intention. We could also show the concept of impedance control for a robot arm and control the robot arm using the decoded joint angles. Finally, this creates remarkable benefits, which would contribute to the realization of ECoG-based prosthetic limbs.

## Figures and Tables

**Table 1:** The cross-validation results of the muscle prediction.

Muscle	*R* ^2^	Mean	Std.
PECM	0.3814 0.3687 0.4381 0.4450 **0.4676**	0.4202	0.0428
DELP	0.0794 0.0967 0.0837 0.0824 **0.1325**	0.0949	0.0220
TRO	0.0664 0.0630 0.0668 **0.0673** 0.0659	0.0659	0.0017
TRA	0.1615 0.1693 0.1783 0.2187 **0.2410**	0.1938	0.0344
BIL	0.3381 0.3960 0.3893 0.3966 **0.4527**	0.3945	0.0406
BRA	0.3512 0.3612 0.3526 0.3616 **0.3658**	0.3585	0.0063
ECR	0.0221 0.0213 0.0148 0.0129 **0.0284**	0.0199	0.0062
EDC	0.5429 0.5433 0.5419 0.5493 **0.5506**	0.5456	0.0040
FDP	**0.6360** 0.6278 0.6350 0.6329 0.6347	0.6333	0.0033
FCU	0.2638 0.2725 0.2751 0.2739 **0.3010**	0.2772	0.0140
APL	0.4419 0.4796 0.5038 0.5189 **0.5533**	0.4995	0.0418
AP	0.5450 0.5426 0.5567 0.5556 **0.6058**	0.5611	0.0257

Muscle	*n*RMSE	Mean	Std.
PECM	0.1482 0.1494 0.1424 **0.1413** 0.1416	0.1446	0.0039
DELP	0.1474 0.1463 0.1476 0.1471 **0.1445**	0.1466	0.0013
TRO	0.1398 **0.1375** 0.1387 0.1383 0.1410	0.1391	0.0014
TRA	0.1637 0.1641 0.1622 0.1628 **0.1614**	0.1628	0.0011
BIL	0.1745 0.1679 0.1692 0.1682 **0.1618**	0.1683	0.0045
BRA	0.1843 0.1832 0.1827 0.1823 **0.1812**	0.1827	0.0011
ECR	**0.1896** 0.1901 0.1903 0.1915 0.1903	0.1904	0.0007
EDC	0.1545 0.1538 0.1547 **0.1533** 0.1534	0.1539	0.0007
FDP	0.1515 **0.1508** 0.1511 0.1512 0.1525	0.1514	0.0007
FCU	0.1857 0.1867 0.1877 0.1875 **0.1650**	0.1825	0.0098
APL	0.1372 0.1335 0.1295 0.1277 **0.1234**	0.1303	0.0053
AP	0.1340 0.1342 0.1359 0.1359 **0.1288**	0.1338	0.0029

**Table 2:** The cross-validation results of the joint angle prediction.

Joint angle	*R* ^2^	Mean	Std.
S1 (abd./add.)	**0.7085** 0.6598 0.6499 0.5474 0.5868	0.6305	0.0635
S2 (Flex./Ext.)	**0.7086** 0.6937 0.6030 0.4869 0.6872	0.6359	0.0929
S3 (rot.)	0.2504 −0.3530 0.1134 −0.4715 **0.2940**	−0.0333	0.3547
E1	**0.6657** 0.6176 0.6455 0.5049 0.5852	0.6038	0.0630

Joint angle	*n*RMSE	Mean	Std.
S1 (abd./add.)	**0.1153** 0.1249 0.1361 0.1465 0.1263	0.1298	0.0119
S2 (flex./ext.)	**0.1284** 0.1390 0.1529 0.1621 0.1412	0.1447	0.0130
S3 (rot.)	**0.1289** 0.1997 0.1601 0.1729 0.1364	0.1596	0.0286
E1	**0.1135** 0.1196 0.1288 0.1429 0.1257	0.1261	0.0111

## Data Availability

The data used to support the findings of this study were supplied by prof. Nishimura and prof. Isa under license and so cannot be made freely available. Requests for access to these data should be made to prof. Nishimura (yukio@nips.ac.jp).

## Supplementary Materials

Supplementary Materials S1_Monkey_Robot_1.wmv.

## References

[1] Wang W., Collinger J. L., Degenhart A. D. (2013). An electrocorticographic brain interface in an individual with tetraplegia. *PLoS One*.

[2] Wolpaw J. R., Mcfarland D. J., Neat G. W., Forneris C. A. (1991). An EEG-based brain-computer interface for cursor control. *Electroencephalography and Clinical Neurophysiology*.

[3] Blankertz B., Dornhege G., Schafer C. (2003). Boosting bit rates and error detection for the classification of fast-paced motor commands based on single-trial EEG analysis. *IEEE Transactions on Neural Systems and Rehabilitation Engineering*.

[4] Waldert S., Preissl H., Demandt E. (2008). Hand movement direction decoded from MEG and EEG. *Journal of Neuroscience*.

[5] Birbaumer N., Ghanayim N., Hinterberger T. (1999). A spelling device for the paralysed. *Nature*.

[6] Pfurtscheller G., Neuper C. (1994). Event-related synchronization of mu rhythm in the EEG over the cortical hand area in man. *Neuroscience Letters*.

[7] Birbaumer N., Cohen L. G. (2007). Brain-computer interfaces: communication and restoration of movement in paralysis. *Journal of Physiology*.

[8] Shindo K., Kawashima K., Ushiba J. (2011). Effects of neurofeedback training with an electroencephalogram-based brain-computer interface for hand paralysis in patients with chronic stroke: a preliminary case series study. *Journal of Rehabilitation Medicine*.

[9] Daneshzand M., Faezipour M., Barkana B. D. (2017). Computational stimulation of the basal Ganglia neurons with cost effective delayed Gaussian waveforms. *Frontiers in Computational Neuroscience*.

[10] Leuthardt E. C., Schalk G., Wolpaw J. R., Ojemann J. G., Moran D. W. (2004). A brain-computer interface using electrocorticographic signals in humans. *Journal of Neural Engineering*.

[11] Chao Z. C., Nagasaka Y., Fujii N. (2010). Long-term asynchronous decoding of arm motion using electrocorticographic signals in monkey. *Frontiers in Neuroengineering*.

[12] Shimoda K., Nagasaka Y., Chao Z. C., Fujii N. (2012). Decoding continuous three-dimensional hand trajectories from epidural electrocorticographic signals in Japanese macaques. *Journal of Neural Engineering*.

[13] Chen C., Watanabe D. H., Nakanishi Y. (2013). Prediction of hand trajectory from electrocorticography signals in primary motor cortex. *PLoS One*.

[14] Chen C., Watanabe D. H., Nakanishi Y. (2014). Decoding grasp force profile from electrocorticography signals in non-human primate sensorimotor cortex. *Neuroscience Research*.

[15] Levine S. P., Huggins J. E., BeMent S. L. (2000). A direct brain interface based on event-related potentials. *IEEE Transactions on Rehabilitation Engineering*.

[16] Pistohl T., Ball T., Schulze-Bonhage A., Aertsen A., Mehring C. (2008). Prediction of arm movement trajectories from ECoG-recordings in humans. *Journal of Neuroscience Methods*.

[17] Pistohl T., Schulze-Bonhage A., Aertsen A., Mehring C., Ball T. (2012). Decoding natural grasp types from human ECoG. *NeuroImage*.

[18] Mehring C., Nawrot M. P., de Oliveira S. C. (2004). Comparing information about arm movement direction in single channels of local and epicortical field potentials from monkey and human motor cortex. *Journal of Physiology-Paris*.

[19] Shin D., Watanabe H., Kambara H. (2012). Prediction of muscle activities from electrocorticograms in primary motor cortex of primates. *PLoS One*.

[20] Nakanishi Y., Yanagisawa T., Shin D. (2013). Prediction of three-dimensional arm trajectories based on ECoG signals recorded from human sensorimotor cortex. *PLoS One*.

[21] Nakanishi Y., Yanagisawa T., Shin D. (2014). Decoding fingertip trajectory from electrocorticographic signals in humans. *Neuroscience Research*.

[22] Nakanishi Y., Yanagisawa T., Shin D. (2017). Mapping ECoG channel contributions to trajectory and muscle activity prediction in human sensorimotor cortex. *Scientific Reports*.

[23] Morrow M. M., Miller L. E. (2003). Prediction of muscle activity by populations of sequentially recorded primary motor cortex neurons. *Journal of Neurophysiology*.

[24] Santucci D. M., Kralik J. D., Lebedev M. A., Nicolelis M. A. L. (2005). Frontal and parietal cortical ensembles predict single-trial muscle activity during reaching movements in primates. *European Journal of Neuroscience*.

[25] Koike Y., Hirose H., Sakurai Y., Iijima T. (2006). Prediction of arm trajectory from a small number of neuron activities in the primary motor cortex. *Neuroscience Research*.

[26] Yoshimura N., Dasalla C. S., Hanakawa T., Sato M. A., Koike Y. (2012). Reconstruction of flexor and extensor muscle activities from electroencephalography cortical currents. *NeuroImage*.

[27] Yoshimura N., Jimura K., Dasalla C. S. (2014). Dissociable neural representations of wrist motor coordinate frames in human motor cortices. *NeuroImage*.

[28] Koike Y., Kawato M. (1995). Estimation of dynamic joint torques and trajectory formation from surface electromyography signals using a neural-network model. *Biological Cybernetics*.

[29] Shin D., Kim J., Koike Y. (2009). A myokinetic arm model for estimating joint torque and stiffness from EMG signals during maintained posture. *Journal of Neurophysiology*.

[30] Sato M. (2001). Online model selection based on the variational bayes. *Neural Computation*.

[31] Osu R., Franklin D. W., Kato H. (2002). Short- and long-term changes in joint co-contraction associated with motor learning as revealed from surface EMG. *Journal of Neurophysiology*.

[32] Heliot R., Orsborn A. L., Ganguly K., Carmena J. M. (2010). System architecture for stiffness control in brain-machine interfaces. *IEEE Transactions on Systems, Man and Cybernetics-Part A: Systems and Humans*.

[33] Kawase T., Kambara H., Koike Y. (2011). A power assist device based on joint equilibrium point estimation from EMG signals. *Journal of Robotics and Mechatronics*.

[34] Osu R., Gomi H. (1999). Multijoint muscle regulation mechanisms examined by measured human arm stiffness and EMG signals. *Journal of Neurophysiology*.

